# Distinct lesion features and underlying mechanisms in patients with acute multiple infarcts in multiple cerebral territories

**DOI:** 10.3389/fneur.2022.1102505

**Published:** 2023-01-16

**Authors:** Yuhui Sha, Guangsong Han, Yuehui Hong, Juanjuan Wu, Mingyu Tang, Yicheng Zhu, Lixin Zhou, Jun Ni

**Affiliations:** State Key Laboratory of Complex Severe and Rare Diseases, Department of Neurology, Peking Union Medical College Hospital, Chinese Academy of Medical Sciences and Peking Union Medical College, Beijing, China

**Keywords:** acute multiple infarcts, diffusion-weighted imaging, etiology, lesion distribution, multiple cerebral territories

## Abstract

**Objective:**

To determine the etiology spectrum and lesion distribution patterns of patients with acute multiple infarcts in multiple cerebral territories (AMIMCT) and provide guidance for treatment and prevention strategies in these patients.

**Methods:**

Patients with acute ischemic stroke diagnosed using diffusion-weighted imaging (DWI) were consecutively included in this study between June 2012 and Apr 2022. AMIMCT was defined as non-contiguous focal lesions located in more than one cerebral territory with acute neurological deficits. We retrospectively analyzed the clinical and imaging characteristics, etiology spectra and underlying mechanisms in patients with and without AMIMCT. Infarct lesion patterns on DWI and their relevance to etiology were further discussed.

**Results:**

A total of 1,213 patients were enrolled, of whom 145 (12%) were diagnosed with AMIMCT. Patients with AMIMCT tended to be younger (*P* = 0.016), more often female (*P* = 0.001), and exhibited less common conventional vascular risk factors (*P* < 0.05) compared to those without AMIMCT. The constitution of the Trial of Org 10,172 in Acute Stroke Treatment classification was significantly different between patients with and without AMIMCT (*P* = 0.000), with a higher proportion of stroke of other determined causes (67.6% vs. 12.4%). For detailed etiologies, autoimmune or hematologic diseases were the most common (26.2%) etiologies of AMIMCT, followed by periprocedural infarcts (15.2%), cardioembolism (12.4%), tumor (12.4%), large artery atherosclerosis (10.3%), and sudden drop in blood pressure (8.3%). Hypercoagulability and systemic hypoperfusion are common underlying mechanisms of AMIMCT. Distinctive lesion distribution patterns were found associated with stroke etiologies and mechanisms in AMIMCT. Most of patients with large artery atherosclerosis (73.3%), autoimmune/hematologic diseases (57.9%) manifested the disease as multiple infarct lesions located in bilateral supratentorial regions. However, 66.7% of cardioembolism and 83.8% of cardiovascular surgery related stroke presented with both supratentorial and infratentorial infarct lesions.

**Conclusion:**

The etiologies and mechanisms of patients with AMIMCT were more complex than those without AMIMCT. The distribution characteristics of infarct lesions might have important implications for the identification of etiology and mechanism in the future, which could further guide and optimize clinical diagnostic strategies.

## Introduction

Data from the Global Burden of Disease Study showed that stroke was the second leading cause of death and the third leading cause of death and disability worldwide (1). According to the National Epidemiological Survey of Stroke in China, the age-standardized stroke prevalence was 1,115 cases per 1,00,000 people (2). Although treatment in the acute phase and secondary prevention have significantly reduced the risk of morbidity and recurrence, these strategies may be less effective for patients with specific types of acute ischemic stroke, which demonstrate more complex mechanisms and deserve more attention.

In terms of infarct lesion distribution, acute ischemic stroke can fall into one of the following categories: single infarct, scattered infarcts in one cerebral territory, and acute multiple infarcts in multiple cerebral territories (AMIMCT). AMIMCT is defined as non-contiguous focal lesions located in more than one cerebral territory with acute neurological deficits (3, 4). Patients with multiple acute cerebral infarcts exhibit higher National Institutes of Health Stroke Scale (NIHSS) score (5), indicating a more severe clinical situation. As the underlying mechanisms are yet to be determined, common therapies are sometimes ineffective, leading to an unsatisfactory clinical outcome with poor prognosis and high recurrence risk. The accurate identification of the etiologies and mechanisms of AMIMCT is the main clinical challenge, which is essential for both acute-phase management and secondary prevention. However, owing to the limited sample size and methodological differences in various studies, data on the frequency and etiology of multiple infarcts are still controversial (6). To date, the etiology classification is mostly based on the Trial of Org 10,172 in Acute Stroke Treatment (TOAST), which is insufficient to guide practical work in these patients.

As for AMIMCT, the management of the current vascular event and the secondary prevention should be implemented aggressively based on the etiology. Diffusion-weighted imaging (DWI) provides sensitive detection of acute ischemic injury within the first few hours (7). Distinctive topographical patterns of multiple acute infarcts indicate different stroke etiologies (3, 8–11). Thus, it is reasonable to infer underlying etiologies *via* lesion distribution characteristics on DWI. Multiple infarcts located in more than one territory are generally believed to be caused by cardioembolism (CE) or systemic factors (4). Roh et al. (6) reported that CE was the most common cause of acute infarcts in both the anterior and posterior circulations. Acute multiple infarcts involving both the anterior and posterior circulations are more frequent in patients with malignant tumors (12). Approximately one out of five patients with multiple infarcts involving three arterial territories are associated with malignancy (13). However, another study showed that large artery atherosclerosis (LAA) was the most common cause in patients with stroke involving the bilateral hemispheres (6). Therefore, the association between the characteristics of infarct lesions and the underlying etiologies requires further investigation.

In this single-center retrospective study of consecutive patients in a large comprehensive hospital in China, we aimed to compare the characteristics of patients with and without AMIMCT and investigate the etiologies and underlying mechanisms of AMIMCT in a more detailed manner. Furthermore, the association between specific etiologies and lesion patterns on DWI was determined, which may help infer the etiology and guide specific treatments in patients with AMIMCT.

## Methods

### Study population

We retrospectively included patients consecutively admitted to Peking Union Medical College Hospital (PUMCH) between June 2012 and Apr 2022. A total of 1,213 patients diagnosed with acute ischemic stroke, defined as sudden onset of neurological deficits with hyperintense DWI lesions on MRI, were included.

This study followed the Strengthening the Reporting of Observational Studies in Epidemiology reporting guidelines. This study was approved by the ethical review board of PUMCH.

### Patient characteristics and definitions

#### Patient characteristics included

1. Demographic data
2. Medical history and vascular risk factors: hypertension, hyperlipidemia (medical history and/or total cholesterol of >5.2 mmol/L and/or low-density lipoprotein-cholesterol of >3.36 mmol/L), diabetes mellitus (medical history and/or an HbA1c level of >6.3%), coronary heart disease, atrial fibrillation (medical history and/or ECG abnormalities), hyperhomocysteinemia (a serum homocysteinemia level of ≥15 mmol/L), stroke, migraine, smoking status, alcohol consumption
3. Family history: a history of cerebrovascular disease in first- and second-degree relatives
4. Previous medication: antiplatelet therapy, statin therapy, anticoagulant therapy, and long-term oral contraceptives
5. Stroke severity: initial NIHSS and modified Ranking Score (mRS) at discharge
6. Neuroimaging features
7. Auxiliary examination results: carotid artery ultrasound (abnormalities: intima thickness, plaque, stenosis, or occlusion), erythrocyte sedimentation rate (ESR, elevated level: >15 mm/h for male and 20 mm/h for female), high-sensitivity acute phase reactants (hsCRP, elevated level: >8.0 mg/L), D-dimer (elevated level: >0.55 ng/mL), infectious disease indicators (human immunodeficiency virus-Ag/Ab, treponema pallidum-Ab, hepatitis C virus-Ab, and hepatitis B-Ag).

Data from the electronic medical records were reviewed. Detailed etiologies were assessed by two experienced neurologists (JN and YHS) based on the clinical and imaging characteristics. The stroke team in PUMCH reviewed the imaging and etiologies together in cases of inconsistencies.

### Imaging sequences and definition of AMIMCT

All MRI was performed at 3.0T. MRI protocol included axial T1-weighted DWI for 2 b-values (b0 and b1000 s/mm^2^) and fluid attenuated inversion recovery. Acute infarct lesions were evaluated using DWI. Vascular examination included CT angiography, magnetic resonance angiography (MRA), transcranial Doppler, and digital subtraction angiography.

**Figure 1:**
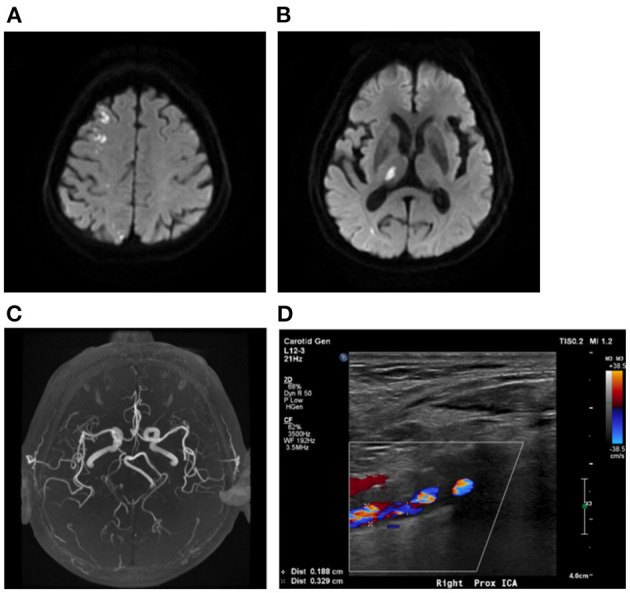
Multiple infarcts caused by variation of large artery. A 75-year-old man presented with weakness in left upper and lower limbs at onset with infarcts located at right frontal lobe and thalamus **(A, B)**. MRA showing right fetal-type posterior artery **(C)**, and the carotid artery ultrasound showing severe stenosis of proximal right ICA **(D)**. MRA, magnetic resonance angiograph; ICA, internal carotid artery.

Patients with AMIMCT were defined as having non-contiguous hyperintense DWI lesions located within multiple cerebral territories, including (1) bilateral anterior circulations, (2) unilateral anterior circulation and posterior circulation and (3) bilateral anterior circulations and posterior circulation (3, 8, 9). Patients with multiple infarcts on DWI, probably caused by congenital vascular variations, were excluded from this study, including the fetal-type posterior cerebral artery (PCA) (Figure 1), recurrent artery of Heubner (supplying inferomedial parts of bilateral caudate heads), artery of Percheron (supplying bilateral medial thalamic nuclei), and unilateral A1 segment aplasia.

### Classification of stroke etiologies

**Figure 2:**
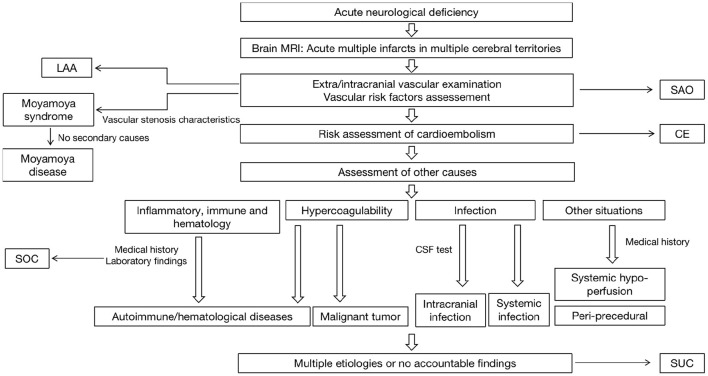
The diagnostic algorithm of AMIMCT in clinical work. LAA, large artery atherosclerosis; SAO, small artery occlusion; CE, cardioembolism; SOC, stroke of other determined causes; SUC, stroke of undetermined causes.

Based on the TOAST classification, detailed stroke etiologies in this study are further classified as follows:

1. LAA
2. Small artery occlusion (SAO)
3. CE
4. Stroke of other determined causes (SOC): (1) autoimmune/hematologic diseases, (2) periprocedural infarction, (3) moyamoya disease, (4) tumor, (5) sudden drop in blood pressure (BP), (6) transient prothrombotic state
5. Stroke of undetermined causes (SUC): (1) two or more etiologies and (2) undetermined etiology (cryptogenic or incomplete examination).
6. Figure 2 showed the Diagnostic algorithm of AMIMCT.

### Statistical analysis

Patient characteristics are presented as median (interquartile range, IQR), considering the skewed distribution. Categorical variables are presented as proportions (*n*, %). We used Student's *t*-test and Mann–Whitney U-test for continuous variables. Pearson's χ^2^ test or Fisher's exact test was used for categorical variables. All tests were two-tailed, and statistical significance was determined at an alpha level of 0.05. Statistical analyses were performed using SPSS (v 28.0).

## Results

### Demographics and baseline characteristics between patients with and without AMIMCT

Among the 1,213 consecutive patients with clinical- and MRI-confirmed acute cerebral infarcts, 145 (12%) met the inclusion criteria for AMIMCT. Table 1 shows the baseline characteristics of patients with and without AMIMCT.

**Table 1:** Baseline characteristics of patients with and without AMIMCT.

	**With AMIMCT *N* = 145**	**Without AMIMCT *N* = 1,068**	**P**
**Demographics**			
Age (median, IQR)	58 (45, 71)	64 (54, 73)	0.016
Male (*n*, %)	77 (53.1%)	716 (67%)	0.001
**Medical history, risk factors and previous medication (** ***n*** **, %)**			
Hypertension	76 (52.4%)	703 (65.8%)	0.002
Coronary heart disease	23 (15.9%)	151 (14.2%)	
Previous stroke history	36 (24.8%)	279 (26.3%)	
Atrial fibrillation	22 (15.2%)	135 (12.6%)	
Diabetes mellitus	44 (30.3%)	441 (41.3%)	0.012
Hyperlipidemia	49 (33.8%)	540 (50.7%)	0.000
Hyperhomocysteinemia	41 (47.7%)	351 (44.6%)	
Smoking status	49 (33.8%)	476 (44.7%)	0.013
Alcohol consumption	26 (17.9%)	326 (30.6%)	0.002
Previous anti-platelet therapy	82 (56.6%)	872 (82%)	0.000
Previous statin therapy	81 (55.9%)	847 (79.5%)	0.000
Previous anticoagulant therapy	31 (21.4%)	103 (9.7%)	0.000
Family history of cerebral vascular disease	21 (14.5%)	224 (21.1%)	
Baseline NIHSS (median, IQR)	5 (2.5, 8)	4 (2, 8)	
mRS (0–1) at discharge (*n*, %)	46 (31.7%)	430 (40.6%)	0.041
**Examination and laboratory test**			
Abnormalities on carotid artery ultrasound (*n*, %)	78 (73.6%)	724 (85.2%)	0.002
Elevated D-Dimer (*n*, %)	101 (73.7%)	420 (43.3%)	0.000
Elevated ESR (*n*, %)	59 (65.6%)	208 (38.4%)	0.000
Elevated hsCRP (*n*, %)	50 (42.4%)	226 (30.1%)	0.008
Positive infectious indicators (*n*, %)	3 (2.3%)	28 (2.9%)	
**TOAST classification (** ***n*** **, %)**			0.000
Large artery atherosclerosis	15 (10.3%)	543 (50.8%)	
Small artery occlusion	2 (1.4%)	194 (18.2%)	
Cardioembolism	18 (12.4%)	100 (9.4%)	
Stroke of other determined cause	98 (67.6%)	132 (12.4%)	
Stroke of undetermined cause	12 (8.3%)	99 (9.3%)	
**Radiological features (** ***n*** **, %)**			0.000
Supratentorial	82 (56.6%)	771 (78.4%)	
Infratentorial	0	178 (18.1%)	
Both supratentorial and infratentorial	63 (43.4%)	34 (3.5%)	

AMIMCT, acute multiple infarcts in multiple cerebral territories; NIHSS, national institutes of health stroke scale; mRS, modified ranking score.

Patients with AMIMCT were younger (58 vs. 64 years; *P* = 0.016) and more often female (46.9 vs. 33%; *P* = 0.001) than those without AMIMCT. Patients with AMIMCT were less likely to have hypertension (52.4 vs. 65.8%; *P* = 0.002), diabetes mellitus (30.3 vs. 41.3%; *P* = 0.012), hyperlipidemia (33.8 vs. 50.7%; *P* = 0.000), smoking habit (33.8 vs. 44.7%; *P* =0.013), alcohol consumption (17.9% vs. 30.6%; *P* = 0.002), and received less antiplatelet (56.6 vs. 82%; *P* = 0.000) and cholesterol-lowering therapy (55.9 vs. 79.5%; *P* = 0.000) compared to those without AMIMCT. However, anticoagulant therapy was more frequent in patients with AMIMCT (21.4 vs. 9.7%; *P* = 0.000) than in those without AMIMCT.

Laboratory tests revealed that lower proportion of abnormalities on carotid artery ultrasound (73.6 vs. 85.2%; *P* = 0.002) and higher levels of D-dimer (73.7 vs. 43.3%; *P* = 0.000), ESR (65.6 vs. 38.4%; *P* = 0.000), and hsCRP (42.4 vs. 30.1%; *P* = 0.008) were found in patients with AMIMCT than in those without AMIMCT.

The proportion of mRS 0–1 at discharge was lower in patients with AMIMCT than in those without AMIMCT (31.7 vs. 40.6%, *P* = 0.041), although there was no statistical difference at the baseline NIHSS. The constitution of the TOAST classification was significantly different between patients with and without AMIMCT (*P* = 0.000), with 67.6 vs. 12.4% SOC and 10.3 vs. 50.8% LAA. The lesion distribution also differed between the two groups (*P* = 0.000).

### Stroke etiologies of patients with AMIMCT

According to the TOAST classification, CE was found in 18 patients, among whom the most common cause was atrial fibrillation (*n* = 13). Other cardiac causes included infective endocarditis and thrombus formation in left ventricle. LAA was observed in 15 patients and SAO in two patients. Other patients were classified as SOC or SUC (*n* = 110).

Of the 110 patients with AMIMCT defined as SOC and SUC, 38 were diagnosed with autoimmune or hematologic disease, including connective tissue disease (CTD)/vasculitis involving the CNS, thrombotic microangiopathy, and Schimke immunoosseous dysplasia. Periprocedural ischemic stroke accounted for 20% of SOC and SUC (*n* = 22). Eighteen patients were classified as having tumor associated stroke, and 12 patients were attributed to a sudden drop in BP, including excessive antihypertensive therapy, septic shock, massive blood loss, and left heart failure. Six patients were identified as transient prothrombotic state, including intracranial infection (*n* = 3), systemic infection (*n* = 2) and eosinophilia (*n* = 1). Among the 3 patients with intracranial infections, two of them had intracranial bacterial infection and one of them underwent viral encephalitis. The acute multiple infarcts occurred 1 to 3 weeks after infection onset. Two patients had moyamoya disease.

Notably, distinct etiologies co-existed in seven patients, among which three patients simultaneously developed atrial fibrillation and a malignant tumor. Other patients who met the standards for LAA also had atrial fibrillation, systemic infection, or rheumatic heart disease. Five patients were classified as having an undetermined etiology because either no reasonable causes were found, or the examination was incomplete. The detailed etiologies of AMIMCT are listed in Table 2.

**Table 2:** Stroke etiologies of AMIMCT.

**Stroke etiologies of AMIMCT**		
**Large artery atherosclerosis**	15	10.3%
**Small artery occlusion**	2	1.4%
**Cardioembolism**	18	12.4%
Atrial fibrillation	13	
Infective endocarditis	4	
Thrombus formation in left ventricle	1	
**Autoimmune/hematologic diseases**	38	26.2%
CTD/Vasculitis involving CNS	33	
SLE/APS	20	
PACNS	1	
Takayasu arteritis	2	
GPA	1	
EGPA	3	
Rheumatoid vasculitis	1	
Behcet's disease	1	
Vasculitis with undetermined reason	3	
Other CTD	1	
Thrombotic microangiopathy	4	
TTP	3	
HUS	1	
Schimke immunoosseous dysplasia	1	
**Peri-procedural infarction**	22	15.2%
Pre-procedural infarction	2	
Intra-procedural infarction	1	
Post-procedural infarction	19	
**Moyamoya disease**	2	1.4%
**Tumor**	18	12.4%
**Sudden drop in blood pressure**	12	8.3%
Excessive antihypertensive therapy	5	
Septic shock	5	
Massive blood loss	1	
Left heart failure	1	
**Transient prothrombotic state**	6	4.1%
Intracranial infection	3	
Systemic infection	2	
Eosinophilia	1	
**Two or more etiologies**	7	4.8%
LAA + Af + systemic infection	1	
LAA + rheumatic heart disease	1	
LAA + Sudden drop in BP	1	
Af + malignant tumor	3	
Surgery + Moyamoya syndrome + malignant tumor	1	
Total	145	

CTD, connective tissue disease; CNS, central nervous system; SLE, systemic lupus erythematosus; APS, antiphospholipid syndrome; GPA, granulomatosis with polyangiitis; EGPA, eosinophilic granulomatosis with polyangiitis; TTP, thrombotic thrombocytopenic purpura; HUS, hemolytic uremic syndrome; LAA, large artery atherosclerosis; Af, atrial fibrillation; BP, blood pressure.

### Association between DWI lesion patterns with detailed etiologies in patients with AMIMCT

**Figure 3:**
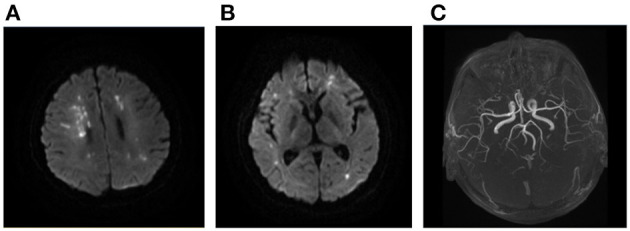
Ischemic lesions related to hypercoagulability. A 22-year-old man with SLE and APS presented with weakness of left upper and lower limbs. Multiple sporadic infarcts were located only at supra-tentorial regions involving bilateral hemispheres **(A, B)**. No large vessel stenosis was found in the MRA **(C)**. SLE, systemic lupus erythematosus; APS, antiphospholipid syndrome; MRA, magnetic resonance angiograph.

**Figure 4:**
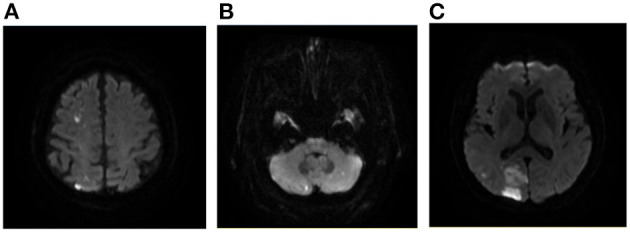
A case of AMIMCT caused by cardiogenic embolism. A 76-year-old man with atrial fibrillation developed left homonymous hemianopia, left upper limb weakness, and ataxia. The lesions involved right frontal, parietal, and occipital lobes and bilateral cerebellar hemispheres **(A–C)**.

**Figure 5:**
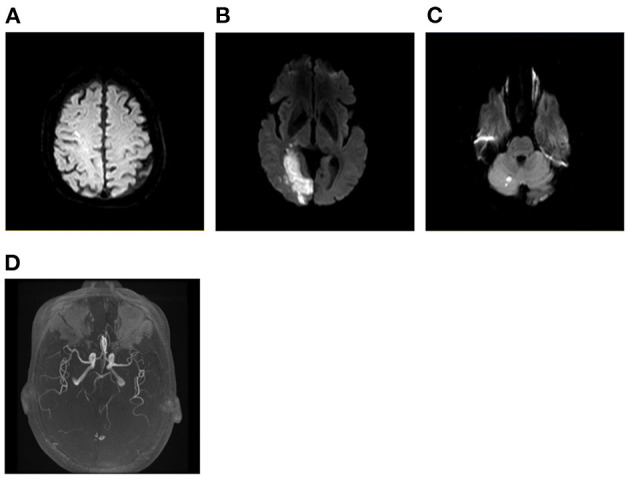
A case of peri-operative infarction. A 72-year-old man presented with left homonymous hemianopia, weakness of left upper and lower limbs, and numbness of left face and limbs after undergoing lung surgery. Infarcts were distributed at right cortex of frontal lobe, right occipital lobe, and right cerebellar hemisphere **(A–C)**. Vascular evaluation showing severe stenosis of right PCA **(D)**. PCA, posterior cerebral artery.

**Figure 6:**
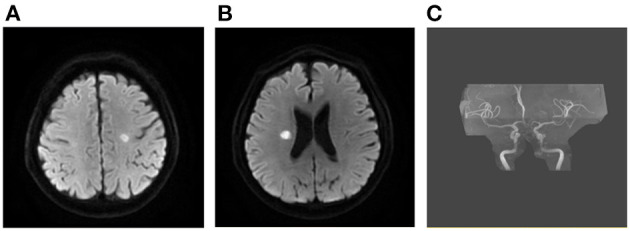
DWI of patients with cerebral small vessel disease. A 51-year-old man had developed left-side central facial palsy, weakness of left limbs, and right hand numbness. MRI showing bilateral supra-tentorial ischemic lesions **(A, B)**. MRA did not show obvious large artery stenosis **(C)**, and no other etiology was found. DWI, diffusion-weighted imaging; MRI, magnetic resonance imaging; MRA, magnetic resonance angiograph.

We investigated the association between DWI lesion patterns and the underlying etiologies in our cohort (Table 3). Seventy-five percent of patients with sudden drop in BP, 73.3% of LAA, 57.9% of autoimmune/hematologic diseases (Figure 3) as well as 55.6% of patients with tumor manifested as multiple infarct lesions located in bilateral supratentorial regions. In contrast, CE was more likely to present with both supratentorial and infratentorial lesions (66.7%) (Figure 4). Similarly, five of six patients undergoing cardiovascular surgery including percutaneous coronary intervention (PCI), aortic arch, and ascending aortic aneurysm surgery were found to have multiple lesions distributed in both supratentorial and infratentorial areas. Patients receiving other kinds of surgeries such as lung surgery may also present with both supratentorial and infratentorial infarctions (Figure 5). Two patients identified as SAO had bilateral deep region infarcts involving bilateral anterior circulations (Figure 6).

**Table 3:** Detailed lesion distribution patterns of AMIMCT with distinct etiologies.

	**Supratentorial**	**Both supratentorial and infratentorial**
LAA	11 (73.3%)	4 (26.7%)
SAO	2 (100%)	0
CE	6 (33.3%)	12 (66.7%) *P* = 0.034
SOC	56 (57.1%)	42 (42.9%)
SUC	7 (58.3%)	5 (41.7%)
Total	82	63
**SOC**		
Autoimmune/hematologic diseases	22 (57.9%)	16 (42.1%)
Peri-procedural infarction	10 (45.5%)	12 (54.5%)
Moyamoya disease	2 (100%)	0
Tumor	10 (55.6%)	8 (44.4%)
Sudden drop in BP	9 (75%)	3 (25%)
Transient prothrombotic state	3 (50%)	3 (50%)

LAA, large artery atherosclerosis; SAO, small artery occlusion; CE, cardioembolism; SOC, stroke of other determined causes; SUC, stroke of undetermined causes; BP, blood pressure.

**Figure 7:**
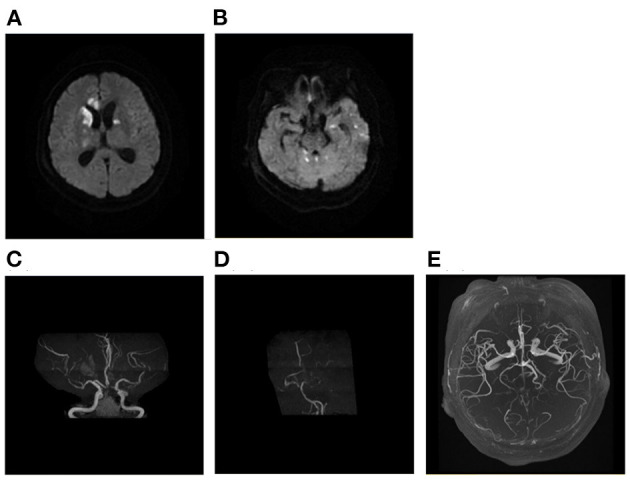
A case of intracranial infection related AMIMCT. A 62-year-old woman with bacterial meningitis had developed infarcts that involved bilateral basal ganglia, corpus callosum, brainstem, and cerebellum **(A, B)**. The MRA showing new bilateral ACA, MCA, and PCA stenosis **(C, D)** compared with MRA performed prior to 1 month **(E)**. AMIMCT, acute multiple infarcts in multiple cerebral territories; MRA, magnetic resonance angiograph; ACA, anterior cerebral artery; MCA, middle cerebral artery.

**Figure 8:**
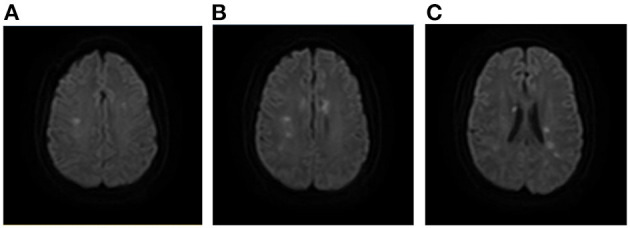
A case of AMIMCT caused by systemic hypo-perfusion. A 37-year-old man had developed severe hypertension and huge fluctuation of blood pressure, which led to multiple stroke. Infarcts were located mainly at the bilateral internal border zones **(A–C)**. AMIMCT, acute multiple infarcts in multiple cerebral territories.

**Figure 9:**
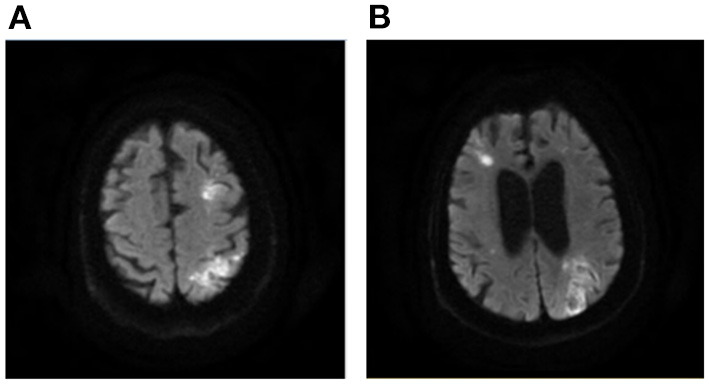
A case of septic shock related AMIMCT. A 77-year-old man who experienced septic shock presented with multiple infarctions located at the bilateral internal border zones and left cortical border zone **(A, B)**. AMIMCT, acute multiple infarcts in multiple cerebral territories.

The patients with intracranial bacterial infection had infarcts located in the bilateral basal ganglia, brainstem and cerebellum, and the affected intracranial arteries were mainly located in the circle of Willis (Figure 7). Patients with rapid antihypertensive treatment tended to present with bilateral internal watershed infarctions (Figure 8) and septic shock-related stroke tended to involve the cortical border zone with a large infarct area (Figure 9).

### Large vessel assessments among patients with AMIMCT identified as SOC in TOAST classification

More than half of the patients with SOC who underwent vascular examination had large artery stenosis, including 42.4% of patients with autoimmune/hematologic diseases, 69.2% of patients with periprocedural infarction, 63.6% of patients with tumor, 60% of patients with a sudden drop in BP, all patients with systemic infection and intracranial infection.

## Discussion

The results of our single-center retrospective study showed that patients with AMIMCT were younger, prone to be female, less likely to have conventional vascular risk factors, usually recipients of anticoagulant treatment, and experienced more severe neurological deficits at discharge compared to those without AMIMCT. The most common etiology of AMIMCT was SOC according to the TOAST classification, followed by CE and LAA. We found that it was common for patients with SOC to coexist with intracranial artery stenosis. The underlying mechanisms of AMIMCT are considered as hypercoagulability, systemic hypoperfusion, embolism, and inflammation, which are more likely to cause infarction when concomitant with large artery stenosis. Further investigation into the relationship between infarct distribution patterns on DWI and etiology indicated that patients with CE and cardiovascular surgery related stroke mostly displayed both supra- and infratentorial lesions, whereas those with sudden drop in BP, large artery atherosclerosis or autoimmune/hematologic diseases related AMIMCT tended to develop lesions only in the supratentorial region. The findings of our study provide significant instructions for etiological diagnosis and targeted secondary prevention in patients with AMIMCT.

The clinical manifestations of patients with AMIMCT were significantly distinctive from those of patients without AMIMCT, which was consistent with previous studies (8, 9), indicating the difference of the underlying mechanisms. In our cohort, the etiological distribution of the TOAST classification of patients with AMIMCT was significantly different from that of patients without AMIMCT, with SOC being the most common cause (67.6%), including autoimmune/hematologic diseases, peri-procedural infarction, tumor related stroke, plunge of BP, moyamoya disease, infection and inflammation-related stroke. CE accounted for 12.4% of our cohort, which was far lower than the data reported in patients with AMIMCT in other countries (which could reach 30%) (5, 8, 9). This was consistent with the previous research evidence that the proportion of CE in Chinese ischemic stroke was lower than that in Western countries (14). Patients in our study were included from a comprehensive hospital specialized in multidisciplinary and difficult diseases; hence, a larger proportion of the study population may have systemic diseases. However, atrial fibrillation still occupied a major part of CE as in previous studies, and other causes including infective endocarditis, myxoma, and thrombus in the left ventricle occurred particularly in young patients with stroke (15), suggesting the importance of etiology-targeted examinations. Moreover, the proportion of large artery atherosclerosis was also lower than the one previously reported, probably due to a strict inclusion criterion described in the methods, since AMIMCT caused by variation of vessels, such as fetal PCA, was excluded in our study.

The etiology distribution of patients with AMIMCT and D-Dimer results in our study suggested that hypercoagulability might be the most important mechanism, including SLE/APS and tumor related hypercoagulability, which led to thrombosis and embolism (16–19). Thus, screening for causes of hypercoagulability based on clinical features in these patients is necessary for subsequent targeted therapy. Besides, systemic hypoperfusion related to a sudden drop in BP is another crucial mechanism caused by various clinical conditions, including excessive antihypertensive therapy, septic shock, massive blood loss, and left heart failure (20). Further vascular assessment in these patients showed that more than half had large artery stenosis, suggesting that intra- and extracranial large artery stenosis are considerable clinical conditions that may elevate vulnerability to stroke (21). Thus, cautious BP reduction and early rehydration are necessary to maintain cerebral perfusion and prevent AMIMCT, especially among patients with large artery stenosis. In particular, we analyzed the mechanisms of peri-procedural infarction. Only eight patients underwent vascular surgery, such as carotid endarterectomy, PCI, the mechanisms of which were considered to be intraoperative hypoperfusion and artery-to-artery embolism (22). Whereas, most of patients with periprocedural infarction received non-vascular surgery, such as pulmonary surgery and orthopedic surgery. Vascular evaluation showed that 69.2% of the patients with peri-procedural infarction had large artery stenosis, indicating that hypoperfusion could not be neglected. Strategies for peri-operative stroke prevention should be made based on large artery assessment before surgery, including avoiding BP fluctuation or maintaining antithrombotic therapy. Inflammation associated with infection, including intracranial and systemic infections, is another important mechanism in patients with AMIMCT. In our study, a patient with intracranial bacterial infection had infarcts involving the bilateral basal ganglia, and the circle of Willis was mainly affected. Infection-related vasculitis may be the major mechanism for artery stenosis or occlusion, which is consistent with previous findings (23). Regardless of hypercoagulability, systemic hypoperfusion, infection or inflammation related AMIMCT, large vessel stenosis was found in a considerable proportion of cases, indicating the potentially essential role of hypoperfusion in the development of AMIMCT. Finally, several etiologies co-existed in some patients (4.8%), such as atrial fibrillation and malignancy, moyamoya disease concomitant with malignancy, suggesting the complexity of AMIMCT with multiple mechanisms involved. Therefore, it is essential to identify all possible etiologies.

We investigated the association between etiology and infarct distribution patterns on DWI. Patients with AMIMCT identified as LAA mainly displayed infarcts in supratentorial regions. However, 66.7% of patients with CE and 83.3% of patients with peri-procedural infarction who underwent cardiovascular surgery had lesions distributed in both supratentorial and infratentorial areas. The results suggest that cardiac-derived or aortic arch-derived emboli should be firstly considered when lesions are located in both the supra- and infra-tentorial regions, which is in line with a previous study (24–27). On the contrary, in most patients with AMIMCT concomitant with autoimmune diseases, the disease tended to present with supratentorial infarctions. Half of the patients with tumor related stroke had infarctions in supratentorial regions, yet the percentage was lower than previous study (28), indicating that the relevance between lesion patterns and tumor related hypercoagulability was not definite. In general, screening for hypercoagulability-related diseases should be performed when multiple infarcts are located only in the supratentorial region, emphasizing tumor screening in the elderly and autoimmune disease evaluation in youth. AMIMCT caused by systemic hypoperfusion have characteristic radiological features. A typical pattern owing to the rapid antihypertensive process tended to be bilateral internal watershed infarctions (21). In contrast, septic shock-related stroke tends to manifest as a cortical border zone infarction with a large infarct area. According to a previous study, cardiogenic or major artery-derived embolism plays a crucial role in the pathogenesis of external border zone infarcts, which are less frequently associated with hemodynamic compromise (29, 30). In our cohort, some patients with septic shock manifested as both internal and external zone infarcts, the mechanism of which was thought to be systemic hypoperfusion concomitant with artery-to-artery embolism (21). Overall, the lesion distribution patterns on DWI could provide implications for detecting etiologies, Consequently, it is of critical importance to understand the radiological features of distinctive mechanisms.

The strength of this study is the relatively large sample with AMIMCT from a high-level general hospital specialized in diagnosis and treatment of complex and rare diseases, facilitating the diagnosis of stroke caused by uncommon reasons. Moreover, the findings of relationship between radiologic features and etiologies are instructive for clinical practice. However, our study has some limitations. There might be a selection bias due to the data from a single-center retrospective study. Additionally, long-term ECG monitoring was not accessible and thus CE could not be identified in some patients. Moreover, complete examinations were not conducted in some severe patients.

## Conclusions

In conclusion, our findings emphasize the complex etiological spectrum of AMIMCT, among which autoimmune or hematologic diseases were the most common, followed by peri-procedural infarcts, CE, and tumor related stroke. Distinctive lesion distribution patterns are associated with stroke etiology in patients with AMIMCT. When it manifests as only the supratentorial region infarcts, hypercoagulability and LAA should be considered first, emphasizing autoimmune diseases in the young and tumor in the elderly. CE and cardiovascular surgery related stroke mostly present with both supratentorial and infratentorial lesions. Systemic hypoperfusion is a crucial mechanism in AMIMCT and tends to present with border zone infarction. More than half of the patients with AMIMCT were found to have large artery stenosis, which increases ischemic susceptibility in those stroke patients. This study provides significant evidence for the targeted management and secondary prevention of AMIMCT.

## Data availability statement

The raw data supporting the conclusions of this article will be made available by the authors, without undue reservation.

## Ethics statement

The studies involving human participants were reviewed and approved by the Ethical Review Board of Peking Union Medical College Hospital. Written informed consent from the participants' legal guardian/next of kin was not required to participate in this study in accordance with the national legislation and the institutional requirements.

## Author contributions

JN, LZ, and YZ contributed to study concept and design. GH, YS, JW, and MT made contributions to data collection and analysis. YS was responsible for manuscript drafting. JN and YH contributed to critical revision of the manuscript for intellectual content. All authors contributed to the article and approved the submitted version.

## References

[1] Global regional and and national burden of stroke and its risk factors 1990–2019: 1990–2019: a systematic analysis for the global burden of disease study 2019. Lancet Neurol. (2021) 20:795–820. 10.1016/s1474-4422(21)00252-0 34487721 PMC8443449

[2] Wu S Wu B Liu M Chen Z Wang W Anderson CS. Stroke in China: advances and challenges in epidemiology, prevention, and management. Lancet Neurol. (2019) 18:394–405. 10.1016/s1474-4422(18)30500-3 30878104

[3] Cho AH Kim JS Jeon SB Kwon SU Lee DH Kang DW. Mechanism of multiple infarcts in multiple cerebral circulations on diffusion-weighted imaging. J Neurol. (2007) 254:924–30. 10.1007/s00415-006-0397-3 17401747

[4] Akhtar T Shahjouei S Zand R. Etiologies of simultaneous cerebral infarcts in multiple arterial territories: a simple literature-based pooled analysis. Neurol India. (2019) 67:692–5. 10.4103/0028-3886.263244 31347536

[5] Novotny V Thomassen L Waje-Andreassen U Naess H. Acute cerebral infarcts in multiple arterial territories associated with cardioembolism. Acta Neurol Scand. (2017) 135:346–51. 10.1111/ane.12606 27109593

[6] Roh JK Kang DW Lee SH Yoon BW Chang KH. Significance of acute multiple brain infarction on diffusion-weighted imaging. Stroke. (2000) 31:688–94. 10.1161/01.str.31.3.688 10700505

[7] Lutsep HL Albers GW DeCrespigny A Kamat GN Marks MP Moseley ME. Clinical utility of diffusion-weighted magnetic resonance imaging in the assessment of ischemic stroke. Ann Neurol. (1997) 41:574–80. 10.1002/ana.410410505 9153518

[8] Depuydt S Sarov M Vandendries C Guedj T Cauquil C Assayag P. Significance of acute multiple infarcts in multiple cerebral circulations on initial diffusion weighted imaging in stroke patients. J Neurol Sci. (2014) 337:151–5. 10.1016/j.jns.2013.11.039 24332593

[9] Sener U Ocek L Ilgezdi I Sahin H Ozcelik M Zorlu Y. Significance of multiple acute ischemic lesions on initial diffusion-weighted imaging in stroke patients and relation of toast classification. Ann Indian Acad Neurol. (2018) 21:197–202. 10.4103/aian.AIAN_487_17 30258262 PMC6137625

[10] Baird AE Lövblad KO Schlaug G Edelman RR Warach S. Multiple acute stroke syndrome: marker of embolic disease? Neurology. (2000) 54:674–8. 10.1212/wnl.54.3.674 10680802

[11] Mustanoja S Putaala J Haapaniemi E Strbian D Kaste M Tatlisumak T. Multiple brain infarcts in young adults: clues for etiologic diagnosis and prognostic impact. Eur J Neurol. (2013) 20:216–22. 10.1111/j.1468-1331.2012.03872.x 23057601

[12] Wang JY Zhang GJ Zhuo SX Wang K Hu XP Zhang H. D-dimer >2785 μg/ml and multiple infarcts ≥3 vascular territories are two characteristics of identifying cancer-associated ischemic stroke patients. Neurol Res. (2018) 40:948–54. 10.1080/01616412.2018.1504179 30317943

[13] Finelli PF Nouh A. Three-Territory DWI acute infarcts: diagnostic value in cancer-associated hypercoagulation stroke (Trousseau syndrome). AJNR Am J Neuroradiol. (2016) 37:2033–6. 10.3174/ajnr.A4846 27365322 PMC7963789

[14] Tsai CF Thomas B Sudlow CL. Epidemiology of stroke and its subtypes in Chinese vs white populations: a systematic review. Neurology. (2013) 81:264–72. 10.1212/WNL.0b013e31829bfde3 23858408 PMC3770160

[15] Stack CA Cole JW A. Diagnostic approach to stroke in young adults. Curr Treat Options Cardiovasc Med. (2017) 19:84. 10.1007/s11936-017-0587-6 28948451

[16] Anzola GP Tincani A Magoni M Spatola L Bonetti A. Neurological involvement in antiphospholipid syndrome: clinical and instrumental evaluation in 21 consecutive cases. Eur J Neurol. (1995) 2:205–9. 10.1111/j.1468-1331.1995.tb00119.x 24283640

[17] Panichpisal K Rozner E Levine SR. The management of stroke in antiphospholipid syndrome. Curr Rheumatol Rep. (2012) 14:99–106. 10.1007/s11926-011-0223-5 22109663

[18] Guraieb-Chahín P Cantú-Brito C Soto-Mota A Guerrero-Torres L Flores-Silva F Chiquete E. Stroke in systemic lupus erythematosus: epidemiology, mechanism, and long-term outcome. Lupus. (2020) 29:437–45. 10.1177/0961203320908947 32151182

[19] Bao L Zhang S Gong X Cui G. Trousseau syndrome related cerebral infarction: clinical manifestations, laboratory findings and radiological features. J Stroke Cerebrovasc Dis. (2020) 29:104891. 10.1016/j.jstrokecerebrovasdis.2020.104891 32807409

[20] D'Amore C Paciaroni M. Border-zone and watershed infarctions. Front Neurol Neurosci. (2012) 30:181–4. 10.1159/000333638 22377891

[21] Mangla R Kolar B Almast J Ekholm SE. Border zone infarcts: pathophysiologic and imaging characteristics. Radiographics. (2011) 31:1201–14. 10.1148/rg.315105014 21918038

[22] Pierik R Uyttenboogaart M Erasmus ME Scheeren TWL van den Bergh WM. Distribution of perioperative stroke in cardiac surgery. Eur J Neurol. (2019) 26:184–90. 10.1111/ene.13793 30152579 PMC6585627

[23] Carod Artal FJ. Clinical management of infectious cerebral vasculitides. Expert Rev Neurother. (2016) 16:205–21. 10.1586/14737175.2015.1134321 26689107

[24] Liu GZ Hu R Peng DT. Chinese expert consensus on the diagnosis of cardiogenic stroke (2019). Chin Med J. (2021) 134:505–7. 10.1097/cm9.0000000000001217 33652457 PMC7929520

[25] Wessels T Wessels C Ellsiepen A Reuter I Trittmacher S Stolz E. Contribution of diffusion-weighted imaging in determination of stroke etiology. AJNR Am J Neuroradiol. (2006) 27:35–9. 16418352 PMC7976056

[26] Kang DW Chalela JA Ezzeddine MA Warach S. Association of ischemic lesion patterns on early diffusion-weighted imaging with TOAST stroke subtypes. Arch Neurol. (2003) 60:1730–4. 10.1001/archneur.60.12.1730 14676047

[27] Bernasconi A Bogousslavsky J Bassetti C Regli F. Multiple acute infarcts in the posterior circulation. J Neurol Neurosurg Psychiatry. (1996) 60:289–96. 10.1136/jnnp.60.3.289 8609506 PMC1073852

[28] Hong CT Tsai LK Jeng JS. Patterns of acute cerebral infarcts in patients with active malignancy using diffusion-weighted imaging. Cerebrovasc Dis. (2009) 28:411–6. 10.1159/000235629 19696480

[29] Nagaratnam N Brakoulias V Ng K. Multiple cerebral infarcts following septic shock. J Clin Neurosci. (2002) 9:473–6. 10.1054/jocn.2001.0987 12217686

[30] El-Gammal TM Bahnasy WS Ragab OAA Al-Malt AM. Cerebral border zone infarction: an etiological study. Egypt J Neurol Psychiatr Neurosurg. (2018) 54:6. 10.1186/s41983-018-0008-0 29780226 PMC5954770

